# A Qualitative Study in Family Units on Organ Donation: Attitude, Influencing Factors and Communication Patterns

**DOI:** 10.3389/ti.2022.10411

**Published:** 2022-03-23

**Authors:** Aijing Luo, Haiyan He, Zehua Xu, Wei Ouyang, Yang Chen, Ke Li, Wenzhao Xie

**Affiliations:** ^1^ The Third Xiangya Hospital of Central South University, Changsha, China; ^2^ Second Xiangya Hospital of Central South University, Changsha, China; ^3^ Key Laboratory of Medical Information Research (Central South University), College of Hunan Province, Changsha, China; ^4^ School of Life Sciences, Central South University, Changsha, China; ^5^ Public Health College of Central South University, Changsha, China

**Keywords:** organ donation, family, attitude, communication patterns, ethics, qualitative research

## Abstract

This study aimed to analyze the attitude, influencing factors and communication patterns of organ donation in Chinses families. We conducted in-depth interviews with 97 participants from 26 families in China from August 2018 to October 2020. Interviews were audio-recorded, transcribed by the researchers. Thematic analysis was used to analyze the data and Nvivo 12 was used to catalog coded data. Thirty-eight participants indicated that they would like to be a donor while the majority were unlikely to donate. Among those who were willing to donate, some disagreed with family members to donate organs. Themes found included attitude, the timing of thinking, taboo and fear, traditional beliefs, ethics and family communication patterns. Lack of knowledge, fear, taboo, some traditional beliefs and mistrust may discourage donation. Altruism and policy which is good for the family seem to encourage donation. We also constructed three family communication patterns to provide a deeper understanding of the family in China. This is the first qualitative study that analyzed attitude, influencing factors and communication patterns based on family units in China mainland. Our findings showed that family comes first in Chinese. We suggest that family-based consent and incentives are more suitable for the Chinese social context.

## Graphical Abstract

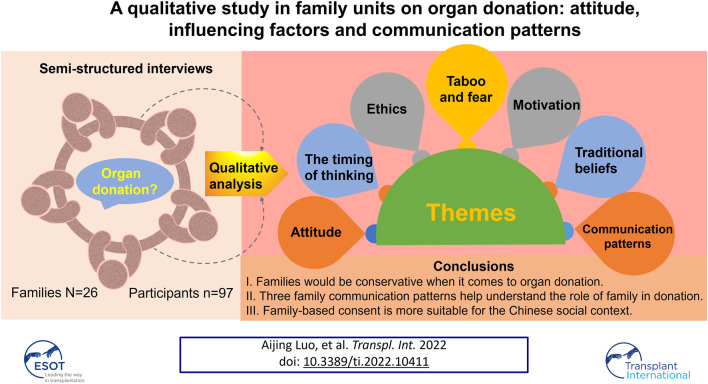

## Background

Organ transplantation has been regarded as a life-saving treatment for patients with organ failure (1). However, demand considerably exceeds the supply of suitable organs made shortage of organs a critical problem worldwide (2,3). For example, there are around 6,000 people on the United Kingdom Transplant Waiting List and over 350 people died while waiting for a transplant in 2019 (4). In the United States, more than 6,000 patients die every year while waiting for a transplant (5). Moreover, organ shortage is particularly serious in China (6), with a donation rate of 3.46 donors per million population (dpmp) compared with the United States (38.0 dpmp) and Spain (37.9 dpmp) (3).

Factors that influence organ donation rate are manifold: organ donation system, legal regulations, cultural beliefs, region, knowledge and attitude toward donation are important factors that affect donation rate worldwide (7–10). Demographic factors such as age, gender, education level, occupation and nationality are also associated with being a donor (11–13). Previous studies have shown that family played an essential role in organ donation (14,15). Researchers collected 1886 questionnaires on organ donation from 11 cities in China, they found that 69.9% of participants considered family consent necessary and 77.1% thought that the view of their family had a great, even decisive, influence on them to decide to become donors (16). By monitoring the public’s discussion about organ donation on a Chinese social media platform (Weibo), among 1,755 posts related to organ donation, most positive posts were ‘‘saluting the organ donors” and most negative posts involved ‘‘fear of the family’s passive medical decision” (17). A study in Taiwan showed that the factors contributing to an aversive preference of cancer patients included the necessity to consider the emotions of family members, traditional perceptions and religious reasons (18). Furthermore, families can overrule the known wishes of the deceased in some countries, such as India, Japan and Canada (19). In Switzerland, although patients had registered as a donor, over 40% of donations were stopped because of family refusal.(20) This phenomenon is particularly common in China. For example, next-to-kin, especially grown-up children, may refuse to carry out the patient’s wish to donate organs after death, because they fear that agreeing to such a wish would not be filial and it would fail to keep the body intact (21). It is therefore of supreme importance to increase family consent on organ donation.

Previous research focused on family’s attitude toward donation (22), family bereavement (23), influencing factors (24), ethical exploration (25) and motivation to donate (26). Many of these studies were conducted in the United States, Australia, Canada and the United Kingdom. Nevertheless, few researchers explored the interaction between family members in organ donation based on Chinese cultural background. Many Chinese were affected by the Confucian cultural norm which states that “It is a responsibility to maintain the physical integrity of the body after death.” Although cremation is currently practiced in China, Chinese funeral custom is inhumation with a whole body. Compared with people who had Confucianism funeral belief, those without the belief were more willing to donate (27). Besides, consent from all immediate family members is required in the practice of organ donation in China. Family communication is important because the opinions of family members on organ donation need to be unified. This research aims to elicit the families’ attitude toward organ donation and how family members interact with each other in China. Family discussion in private settings improves family experience (28). Hence, we explored the family attitude, influencing factors and communication patterns on organ donation by conducting interviews with family units.

## Methods

### Research Design

Qualitative research can capture human emotions and perceptions hidden behind their experiences and offers complementary information to that uncovered by quantitative surveys (12). Therefore, we chose a qualitative method to facilitate an in-depth exploration of family attitude, influencing factors and communication patterns toward organ donation using a semi-structured interview schedule. Interview questions were categorized into three domains: 1) understand the thoughts and attitude to organ donation, 2) confidence in fair distribution of organs, 3) family communication (See Table 1).

**TABLE 1:** Interview guide.

Thoughts and attitude toward organ donation
Have you heard about organ donation?
Is talking about organ donation taboo for you? Why?
What do you think of organ donation?
Cremation is implemented in China. It is better to donate organs to save a life rather than being burned after death. What do you think?
**Confidence in fair distribution of organs**
What makes you want to be a donor/not to be a donor?
Do you believe that organs can be distributed fairly and justly? Why?
What do you think about brain death?
**Family communication**
What do you think about signing the donor card? For example, sign up as a donor when you get your driver’s license?
Will you communicate with your next-to-kin before you make a decision?
Whose opinion would influence you most?
Will you agree with your family members (especially your children/ parents/ couples) if they would like to be a donor? Why?
Will you consider being a donor if donor families would have priority in organ distribution when needed?

### Participants

We have released a recruitment notice on our laboratory’s official website. The inclusion criteria for the volunteers were as follows: 1) above the age of 18, 2) good at communication, 3) would like to talk about organ donation with families, 4) agreed to participate in the interviews. The sampling period lasted from August 2018 to January 2020, a total of 52 volunteers would like to participate in this project. We trained each volunteer in qualitative interview skills to ensure the quality of the experiment. During this period, nine volunteers dropped out of the project for personal reasons, seven were unable to conduct qualitative interviews skillfully. Thus, 36 volunteers conducted semi-structured interviews according to the outline with all their families together face to face or through online video. Family members include but are not limited to parents, spouses, grandparents, uncles and aunts, etc.

We stopped recruiting volunteers and family interviews once we reached theoretical saturation, themes and trends were well developed, and no new concepts or structures emerged (29). Finally, twenty-six families, 97 participants completed the interview and each interview was audio recorded. Ten volunteers failed because family members were reluctant to talk about organ donation. Table 2 shows the demographic characteristics of the participants.

**TABLE 2:** Demographic data of participants.

Characteristic	*n* (%)
Gender	
Male	34 (35)
Female	62 (65)
Age group	
18–30	37 (38)
31–60	56 (58)
61+	4 (4)
Education	
Junior high school and below	20 (21)
Trade school	2 (2)
High school diploma or equivalent	16 (16)
Some college	12 (12)
Bachelor’s degree	18 (19)
Master’s degree	29 (30)
Household income, *N* = 26	
<15,000	9 (35)
15,000–30,000	11 (42)
30,000–45,000	2 (8)
>45,000	4 (15)
Relationship with volunteers	
Volunteers	26 (27)
Parents	42 (43)
Spouse	4 (4)
Grandparents	4 (4)
Brothers/Sisters	10 (11)
Cousins	5 (5)
Uncles/Aunts	3 (3)
Parents-in-law	2 (2)
Other	1 (1)

### Analysis

Interview recordings were transcribed verbatim by the researchers who searched for concepts, themes, and ideas. We performed a qualitative content analysis on these transcripts (30) and used NVivo software (QSR, Version 12) to catalog coded data. At least three coders met regularly to adjudicate differences in codes and discuss emerging themes for higher-level analysis with a focus on content related to family communication.

### Ethics

All participants gave their written consent and were aware that they had the right to withdraw from the project without giving a reason. We promised that the interview content will only be used for scientific research and ensure the privacy of participants. The study was approved by the IRB of the Third Xiangya Hospital, Central South University.

## Results

Key findings on the seven main themes, “attitude to organ donation in family,” “timing of thinking,” “motivation,” “fear and taboo,” “body intact,” “fair and trust,” “family communication patterns” are shown in Table 3.

**TABLE 3:** Themes and representative quotations.

Theme	Subtheme	Representative quotations
Attitude		“I would like to register as an organ donor. but I don’t want my family member to donate. Em…I don’t want them to lose any part of their body.” (N10, mother, 46 y)
		“I can donate my organs when deceased. But I’m afraid if my next to kin missing a part of their body.” (N18, volunteer, 24 y)
Timing of thinking	Seriously ill or facing death	“If I was ill and life is irretrievable, I will consider donation according to the actual situation.” (N2, aunt, 42 y)
	The media	“I once saw a video about organ donation. I vaguely remembered that a baby was crying all the time. However, the baby stopped crying when a man who looked very rough held him. The reason seems to be that the heart of that man was donated by the baby’s mother. The baby didn’t cry when he heard the heartbeat just like his mother. Since I became a mother, I think if my organs can help people in need and continue in their lives, I feel as if I am still in this world and my children can still feel my existence.” (N14, sister, 32 y)
	Sympathy	“You will know how painful they (people who on the waiting list) were when you came to the hospital. Especially seeing a child with organ failure lying on the bed. Just one organ can save and change their lives. I would like to donate my organ after death if someone needed.” (N8, sister, 27 y)
Motivation	Altruism	“I think it’s noble to donate organs and save people’s lives. “(N11, volunteer, 24 y)
		“Organ donation is a good deed that benefits others and society. “(N26, father, 49 y)
	Usefulness	“If the donated organs can be used properly, that can yet be regarded as a continuation of life, make the best use of organs. “(N5, volunteer, 25 y)
		“It’s a waste that being buried or burn after death. It is better to donate to save someone’s life.” (N21, father-in-law, 64 y)
	Good for Family	“I can sacrifice for my family.” (N3, mother, 51 y)
		“If my family members need a transplant and have priority rights of organ, I will be the first one to sign it. “(N4, mother, 46 y)
		“I would not hesitate to do it if it is good for my family.” (N12, cousin, 20 y)
Taboo and fear	Taboo	“It’s inauspicious to talk about it (organ donation), a taboo, I do not like to hear it. “(N7, mother, 49 y)
		“When it comes to organ donation, I will think of death, which makes me sad.” (N5, mother, 49 y)
	A bad omen	“I wouldn’t sign it. It’s like an omen. I don’t like it. “(N5, mother, 49 y)
		“If I sign this thing, I always feel that I have to remind myself from time to time when I drive. That put an inexplicable pressure on me.” (N10, sister, 28 y)
	Fear	“Organs are donated when people die unnaturally.” (N11, father, 45 y)
		“I was so scared to have my organs cut off after death. “(N17, mother, 54 y)
Traditional beliefs	Filial piety	“Your hair and skin are received from your parents. Keeping the body intact is a form of filial piety.” (N2, aunt, 42 y)
		“From ancient times to the present, even if a person died, he should have a complete body.” (N12, father, 51 y)
	Metempsychosis	“I believe in reincarnation. For example, If I donate my cornea, I would be blind next life.” (N1, mother, 49 y)
		“What if I had a heart or kidney problem? What if I lose my arm or leg the next life?” (N6, mother, 55 y)
Ethics	Fairness	“But I think the system and the supervision are not perfect. If I donate my organ, who will use the organ? How much is the charge? Is it reasonable? Anyway, at least now I don’t think it’s fair.” (N14, sister, 32 y)
		“If the rich get sick, they don’t have to wait at all. It seems that they can transplant as long as they are matched. It’s obvious that so many people need organs, but the rich can change one after another. “(N18, volunteer, 24 y)
	Mistrust	“I’ve heard that someone was diagnosed to be brain-dead, and later came back to life. Doctor may misdiagnose” (N3, father, 54 y)
		“I don’t trust the doctor’s brain death diagnosis. What if there’s a miracle?” (N7, volunteer, 24 y)
		“What if my organs are donated and the bad guys illegally make huge profits? Isn’t that against my original intention? I am afraid.” (N14, sister 32 y)
Three family communication patterns	The whole family participate actively	“Organ donation is not just an individual matter, but a whole family matter.” (N7, father, 49 y)
		“You should ask the family for advice first! You cannot decide (organ donation) by yourself.” (N24, brother, 24 y)
		“I’ll ask the whole family for advice, and if they say no. I will not sign it.” (N4, young brother, 19 y)
		“Because organ donation is too important to decide by yourself.” I would ask my wife and parents for advice. (N9, brother, 30 y)
	Family makes the decision for me	“Whatever, I don’t care. It depends on you (son or daughter). After we both die, you can do whatever you want.” (N2, grandmother, 68 y)
		“It depends on my son and daughter. If they agree (donation), then I will agree. “(N4, mother, 46 y)
	Make my own decision	“If I learned more about organ donation, maybe I will sign the donor card. I don’t need to ask my family for advice. I can decide on my own.” (N16, sister, 26 y)
		“If something bad happened to me, I may willing to be a donor. I can make my own decision.” (N16, volunteer, 34 y)
		“I don’t have to ask my family for permission. It’s just like donating blood.” (N21, husband, 32 y)

### Attitude Toward Organ Donation in the Family

We enrolled 26 families, 97 family members in interviews based on family units. Twenty-six families were numbered as N1, N2, N3……N25, N26. Respondents included grandparents, parents, spouses, brothers and sisters, cousins and uncles, etc. Most respondents have heard about organ donation. They learned about organ donation knowledge mainly through TV, the internet, school and friends. According to the interview, we counted the attitude of family members towards organ donation (See Table 4).

**TABLE 4:** The attitude of family members toward organ donation.

Consent to families donation	Personal attitude toward organ donation			
	Willing to donate *n* = 38	Unwilling to donate *n* = 57	Not applicable *n* = 2	Total *n* = 97
Yes	24	2	0	26
Respect theirs wishes	6	14	0	20
NO	8	37	0	45
Not applicable	0	4	2	6

Of 97 participants, 38 expressed their willingness to donate when death is inevitable. However, majority of the participants refused to sign up as a donor. Moreover, there was a great possibility that they would prevent relatives from organ donation. We also found an interesting point, some participants would like to be a donor, but they cannot accept their next-to-kin (especially their parents and children) donate organs. For example, the mother of the N10 family would like to be a donor herself, while she did not want her children or husband to donate organs. The volunteer of the N18 family indicated that she could donate her organ, but she thought it’s unacceptable to donate her parents’ organ.

### The Timing of Thinking

Many of our respondents expressed that they have heard about organ donation. However, few of them talked about it with family members or ever thought to be a donor. Many knew little about donation and felt that organ donation is far behind their lives.

#### Seriously Ill or Facing Death

Many participants indicated that they would consider organ donation when they suffered from an incurable disease or a traffic accident.

#### The Media

Media, which includes television, radio, magazine, and the internet, is important access that people know more about organ donation. Some participants have thought to be a donor because they were deeply touched by the relevant documentaries and public service advertising.

#### Sympathy

Some participants worked at the hospital, they often came into contact with patients with end-stage diseases. They knew how desperately these patients need transplants. Just one organ can save a life and a family. They seemed more likely to donate because of sympathy.

### Motivation

#### Altruism

Altruism is the main factor that participants would like to be a donor. Being able to help someone else is a positive reason for participants to support organ donation. Some participants believed that organ donation is meaningful and can enrich their lives. They can save people who are seriously ill and contribute to society through organ donation.

#### Usefulness

Some agreed that people are turned to ashes after death, it is better to donate organs to help those who need them. Families declared that organs were precious and cherished, they had little worth to the donor but can prolong the lives of others.

#### Good for Family

The family occupies a very important place in Chinese. Many indicated that if families had priority in organ distribution when needed, this will promote their willingness to donate. While for those who had less willing to donate, they would consider registering as an organ donor for their families.

### Taboo and Fear

#### Taboo

Ten volunteers failed to conduct the interview because family members refused to talk about organ donation or they felt uncomfortable during the deep discussion about family member’s donation. They thought it was a taboo topic to them. When talking about organ donation, some expressed that they could not help thinking of bereavement, which made them feel anguished.

#### A Bad Omen

Signing the donor card when getting the driver’s license makes many respondents feel uncomfortable. The participants were more or less superstitious, they would be anxious about something bad will happen to them and their families, and this caused psychological stress on them. Moreover, they did not want their families to worry about them because of signing as a donor.

#### Fear

Including fear of mortality, fear of being separated after death, and fear of unnatural death. Some participants refused donation because they thought that only unnatural death would donate organs.

### Traditional Beliefs

#### Filial Piety

Families who were affected by Confucian culture believed that one should keep the body intact even when they died. That is one of the reasons why people refused the donation. There is an old saying in china that goes “filial piety is the foundation of all virtues.” Thus, some participants who support organ donation may refuse their parent’s organ donation. Because they do not want to be unfilial.

#### Metempsychosis

In China’s unique traditional cultural background, many believed in metempsychosis which may be influenced by Buddhism. They think that the soul will reincarnate after death. Therefore, some mentioned that if the body is incomplete, they would be disabled the next life.

### Ethics

#### Fairness

Many worried about the fairness of the distribution of organs. They indicated that the poor may not be able to afford to transplant operation, so only the rich could have a transplant. Besides, they worried whether the regulatory systems can protect the donor’s rights and interests.

#### Mistrust

Most believed in doctor’s diagnosis of brain death because it is scientifically validated. However, some were skeptical. For one thing, they questioned the scientificity of the brain death diagnosis and wondered if brain-dead people were really dead. For another thing, they were afraid that doctors may not try their best to save them if they signed up as a donor.

### Three Communication Patterns of Families

#### Pattern 1: The Whole Family Participates Actively

Families believed that organ donation is a big deal that everyone should participate to make the decision. In this situation, the willingness of organ donation was greatly affected by the family. If the elders in the family were positive about organ donation, it seems that children would be more likely to accept donation. If someone in the family disagreed, donation was unlikely to succeed. Because they have to consider the opinions and feelings of their families.

#### Pattern 2: Family Makes Decisions for Me

This situation usually happened to the elderly who relied on their sons and daughters. Few Chinese people made wills, although they knew they were responsible for their bodies, they indicated that donate or not is up to posterity.

#### Pattern 3: Make My Own Decision

Some who are not willing to donate may not communicate or ask for advice from family. Three people mentioned that he/she would not consult the family, he/she could make the decision themselves.

## Discussion

This study conducted in-depth qualitative research to analyze the attitude, influencing factors and communication patterns on donation in family units. Most families have never discussed this serious topic with next to kin before and their attitude varied. Limited knowledge, motivation, traditional beliefs, especially family attitude have a great influence on their decision. We constructed three family communication patterns according to the analysis.

There is an interesting point that has never been found in previous studies. Some would like to be a donor while he/she would not agree their next to kin to donate. In our study, thirty-eight indicated that they would like to a donor. However, eight of them couldn’t accept their family donate organs. Besides, people who had little willingness to donate may sign up the donor card if their family could have priority to transplant when needed. All these showed that family comes first in most Chinese, sometimes one may sacrifice for the family. Besides, it seems that filial piety is not only an obstacle to organ donation but also promotes organ donation.

We found the factors from different aspects that influence the family’s decision. Limited knowledge, lack of family discussion, some traditional beliefs and mistrust may discourage donation. Altruism and family support are likely to encourage donation. This is consistent with previous research results (7,31–33). Many refused to talk about organ donation because it is a serious topic that makes them uncomfortable. Some believed that their relatives would not be supportive, because of an excessive number of family members, consensus could not be achieved (18). Besides, in Chinese special culture, we also found some factors that have never been discovered before. For example, people thought that sign up the donor card when healthy may be a bad omen. Some affected by Buddhism believed in metempsychosis. Therefore, they insisted on keeping the body wholeness after death. Overwhelmingly, the imperfect regulatory systems and mistrust made many families refused to sign up the donor card even if they have a strong willingness to donate. People worried that donated organs may be used in improper trading (17). Our research showed that there are many potential donors in public. Thus, regulatory measures are needed to ensure that the rights and interests of donors would be well protected and everyone has an equal right to obtain organs.

Obviously, family opinion played a vital role in the successful donation (34). Previous studies often simply described the decision-making model as family centered (35). Ya-Ping Lin had constructed 3 patterns of communication and decision-making processes in living donor liver transplantation among Tawanese (26). However, we established three family communication patterns that apply to the public in China mainland based on varying family structures, relationships, personal attitude and traditional beliefs. We encouraged communication pattern 1 that the whole family participates actively in the discussion. This could help understand the family’s true will on organ donation and avoid the dilemma of disagreement when facing donation. The implementation of organ donation in China also requires the consensus of family members. Communication pattern 2 is not uncommon among the old. They have less willing to talk about organ donation with family and insist that their sons or daughters would decide for them. While some family with communication pattern 3 would make their own decision without discussion. They believed they do not need to talk about organ donation as they are responsible for their body. Understanding family communication patterns and influencing factors is vital for the policymaker to make perfect law. We suggest that donation regulations need to focus on families and formulate relevant preferential policies based on families.

The previous study also found that family discussion of organ donation was positively related to the attitude toward deceased organ donation (36). However, some family members refused to talk about this topic mainly because of some traditional beliefs and misunderstandings of organ donation. Thus, promoting the knowledge of organ donation and raising public awareness is necessary for improving family communication. Organ donation is regarded as the “gift-of-life” and an act of great love. Confucianism considers physical integrity as a form of filial piety, however, the core of Confucianism emphasizes “ren,” which means benevolence (35). Therefore, organ donation can be promoted based on “ren.” Social media, such as Weibo and WeChat, which were widely used in China (37, 38). OPO should make full use of the internet to share organ donation stories and awaken the heart of benevolence. Besides, publicize knowledge related to organ donation and transplantation would be helpful to dispel misunderstandings of donation. We also recommend that knowledge of organ donation can be added to school education to increase acceptance among the young.

This study has several limitations: a recruitment notice was issued through our laboratory website, we also repost it on our social platforms. In fact, organ donation is rarely talked about in daily life. Therefore, this may not have been a true census sample as we have only invited people who were interested in organ donation. Private discussions among family members enabled participants to better express their true thoughts on organ donation. Thus, we conducted qualitative interview training for volunteers. However, it is time-consuming to train the volunteers and to collect the data, that is the reason why our study lasted for so long. Besides, it is undeniable that controlling the quality of the family interview is not easy because researchers did not participate in the interview process.

## Conclusion

Our study provided a deeper understanding of attitude, influencing factors and communication patterns in families on organ donation in China. We found that families would be conservative when it comes to organ donation. Limited knowledge, fear, some traditional beliefs and mistrust would discourage donation. Based on the analysis, this research provides insight into the family communication on donation. Family always comes first in Chinese society. We suggest that family-based consent and incentives are more suitable for the Chinese social context. Regulatory measures in the process of organ procurement and distribution should be strengthened to protect the interests of donors and increase public trust. Social media are recommended to dispel misunderstand of donation and improve the public’s acceptance of organ donation.

## Data Availability

The data analyzed during this study are available from the corresponding author on reasonable request.
